# A comparison of standard and compositional data analysis in studies addressing group differences in sedentary behavior and physical activity

**DOI:** 10.1186/s12966-018-0685-1

**Published:** 2018-06-15

**Authors:** Nidhi Gupta, Svend Erik Mathiassen, Glòria Mateu-Figueras, Marina Heiden, David M. Hallman, Marie Birk Jørgensen, Andreas Holtermann

**Affiliations:** 1 National Research Centre for the Working Environment, Lersø Parkallé 105, 2100 Copenhagen, Denmark; 2 Centre for Musculoskeletal Research, Department of Occupational and Public Health Sciences, University of Gävle, Kungsbäcksvägen 47, 801 76 Gävle, Sweden; 3 Department of Computer Science, Applied Mathematics and Statistics, University of Girona, Campus Montilivi, Edifici P-IV, 17004 Girona, Spain; 4 Department of Sports Science and Clinical Biomechanics, University of Southern Denmark, Campusvej 55, 5230 Odense M, Denmark

**Keywords:** CoDA, Accelerometry, CoDA-based MANOVA, Isometric log-ratio, Sex, Age

## Abstract

**Background:**

Data on time spent in physical activity, sedentary behavior and sleep during a day is compositional in nature, i.e. they add up to a constant value. Compositional data have fundamentally different properties from unconstrained data in *real* space, and require other analytical procedures, referred to as compositional data analysis (CoDA). Most physical activity and sedentary behavior studies, however, still apply analytical procedures adapted to data in real space, which can lead to misleading results. The present study describes a comparison of time spent sedentary and in physical activity between age groups and sexes, and investigates the extent to which results obtained by CoDA differ from those obtained using standard analytical procedures.

**Methods:**

Time spent sedentary, standing, and in physical activity (walking/running/stair climbing/cycling) during work and leisure was determined for 1–4 days among 677 blue-collar workers using accelerometry. Differences between sexes and age groups were tested using MANOVA, using both a standard and a CoDA approach based on isometric log-ratio transformed data.

**Results:**

When determining differences between sexes for different activities time at work, the effect size using standard analysis (η^2^ = 0.045, *p* < 0.001) was 15% smaller than that obtained with CoDA (η^2^ = 0.052, *p* < 0.001), although both approaches suggested a statistically significant difference. When determining corresponding differences between age groups, CoDA resulted in a 60% larger, and significant, effect size (η^2^ = 0.012, *p* = 0.02) than that obtained with the standard approach (η^2^ = 0.008, *p* = 0.07). During leisure, results based on standard (age; η^2^ = 0.007, *p* = 0.09; sex; η^2^ = 0.052, *p* < 0.001) and CoDA (age; η^2^ = 0.007, *p* = 0.09; sex; η^2^ = 0.051, *p* < 0.001) analyses were similar.

**Conclusion:**

Results and, hence, inferences concerning age and sex-based differences in time spent sedentary and in physical activity at work differed between CoDA and standard analysis. We encourage researchers to use CoDA in similar studies, to adequately account for the compositional nature of data on physical activity and sedentary behavior.

## Background

The health benefits of being physically active are numerous, while sedentary behavior has emerged as a potential health hazard [1, 2]. Both insufficient physical activity and excessive sedentary behavior appear to be associated with an increased risk of coronary heart disease, type 2 diabetes mellitus, and cancer [3–5].

Among various factors, age and sex are two potentially important determinants of sedentary behavior and physical activity [6–9]. For instance, men tend to be more physically active than women [6], and physical activity tends to decrease with age [6].

A majority of studies, including those investigating differences between sexes and age groups in physical activity and sedentary behavior, have used a *standard analysis* approach in which the time spent in each behavior, e.g. time spent sedentary within a day, is treated without consideration to the inherent dependency of time spent in all behaviors occuring within that day. If the time spent in one behavior is changed, it will inevitably influence the time in other behaviors within that day. Data with this inherent dependency in the sense that they add up to a constant sum are *constrained or compositional* [10, 11].

A *standard* multivariate statistical approach for analyzing time spent in different behaviors within a day fails to account for this constrained property of data [12–14]. A set of procedures has been developed to handle compositional data, i.e. *Compositional Data Analysis* (CoDA [10]) which has only recently received attention in studies of sedentary behavior and physical activity [14–20]. One of these studies compared results obtained using standard and CoDA approach, in an investigation of associations between time spent in different behaviors within a day and various health indicators [16]. The study found that associations were different when standard analyses were used, compared to CoDA. No previous study has explicitly investigated the extent to which the results of comparisons between sexes and age groups in time spent in various behaviors during a day depend on whether the analysis was performed using CoDA or a standard approach.

Thus, the present study compared sedentary behavior and physical activity during working days between sexes and age groups, with specific emphasis on differences in results obtained with standard and CoDA approaches.

## Methods

The study was based on cross-sectional baseline data from the Danish PHysical ACTivity cohort with Objective measurements (DPHACTO; c.f., [21]). Data were collected between spring 2012 and spring 2013 at 15 Danish workplaces in three different occupational sectors, i.e. cleaning, transport, and manufacturing. In total, 2107 eligible workers, recruited in collaboration with a large labor union, were invited to participate in the study. Workers were excluded if they had a white-collar job, were pregnant, had a fever, or had an allergy to adhesives.

### Data collection

Participants filled-in a web-based questionnaire and were equipped with an Actigraph accelerometer (Actigraph GT3X+, Florida, USA) placed on the right thigh [22] for four consecutive days (4 × 24 hours), including at least two working days [23]. On the measurement days, the workers were asked to complete a paper-based diary, noting their working hours, time in bed (i.e. the times going to bed and getting out of bed), and non-wear time. They also noted the time of a reference measurement (ie., standing in an upright position for 15 s) performed to allow a coordinate transformation between the axis of the accelerometer and the orientation of the thigh [24]. Instructions to the workers are detailed in previous publications [23, 25, 26].

#### Accelerometer-based measurements of movement behaviors within a day

The amounts of time spent in various behaviors (sedentary, standing, and physical activity (PA)) were identified from the accelerometer recordings using the MATLAB program Acti4 [22, 24, 26]. The Acti4 program has a high sensitivity (>94%) and specificity (>99%) in identifying body postures (sitting and lying) and different physical activities (i.e., standing, walking, running, cycling, and stair climbing) during semi-standardized conditions [22]. Periods spent walking, stair-climbing, running, and cycling were merged to total PA time category.

All non-working days, non-wear periods and bedtime periods were excluded according to previously reported criteria [25, 26]. Workers were included in further analyses only if they had at least one measured day with a valid recording of a work and leisure period. A definition of valid work and leisure period is explained elsewhere [25, 26]. Work was defined as the self-reported hours spent in the primary occupation, and leisure time was defined as the remaining time, except for time in bed. The amounts of time spent in sedentary behavior, standing, and in PA were expressed relative to the total wear time for work and leisure separately.

#### Age and sex

Age and sex were retreived from the unique Danish civil registration number. Workers were categorized into two age groups: younger (≤45 years) and older (>45 years).

### Data processing and statistical analysis

Differences between sexes and age groups in the amounts of time spent in various movement behaviors were analyzed separately for work and leisure. Each comparison was performed using both standard and CoDA analytical approaches.

#### Compositional approach

In CoDA, the compositional data, which lies in a simplex dataspace, can first be mapped to the real space by transforming the absolute values in the composition, i.e. the compositional vector, into sets of log-ratios [13]. The log-ratio transformation leads to data that are not constrained and can take any real value between −∞ and +∞. Several algorithms for log-transformation of compositional data have been proposed [12, 27, 28]. After some types of log-ratio transformations, such as the isometric log-ratio (*ilr*) transformation, data can be processed and analyzed using any *standard* statistical technique that is valid under the conditions and assumptions applying to data in real space.

To investigate differences between sexes and age groups, we performed CoDA in the following steps adapted from previous research [29]:

- **CoDA-based descriptives**. Compositional means were calculated by normalizing the geometric means of all movement behaviors so as to add up to 100%. Bar plots of geometric means (Appendix 2) were used to illustrate proportions of the time spent in each behavior, stratified by sex and age group [29] (cf. Appendix 1 for how to make these plots). Variability in the data, in terms of variability of each behavior relative to the variability of other behaviors, and the total variance of the whole composition, is described in Appendix 3 through a variation matrix [11, 16] within each domain.
- **Log-ratio data transformation**. We selected the *ilr* data transformation rather than other log-transforms such as additive or centered [30] because the *ilr* transformation preserves all metric properties of data and results in coordinates with a non-singular covariance matrix [27]. Specifically, with an *ilr* transformation, data in a simplex with three parts (i.e.,, sedentary, standing and PA) are expressed in real space by two log-ratio coordinates [29]:

1 $$ {\mathrm{ilr}}_1=\sqrt{\frac{2}{3}\ }\mathit{\ln}\frac{(sedentary)}{{\left( stand\times PA\right)}^{\raisebox{1ex}{$1$}\!\left/ \!\raisebox{-1ex}{$2$}\right.}} $$

2 $$ {\mathrm{ilr}}_2=\sqrt{\frac{1}{2}\ }\mathit{\ln}\frac{(stand)}{(PA)} $$

Thus, *ilr*_*1*_ expresses the ratio of sedentary time to time in all other (non-sedentary) behaviors, while *ilr*_*2*_ considers the ratio of standing time to time in PA, i.e. the relative occurrence of the two movement behaviors “within” the non-sedentary class.

Then, multivariate analysis of variance (MANOVA) was applied to the *ilr*-transformed data to determine the separate main effects of sex and age (as independent variables) on both *ilr* log ratios together (dependent variables), using the partial eta squared (η^2^) as a measure of effect size, and the corresponding *p*-value as a metric for evaluating statistical significance. To understand and complete the results of the multivariate tests, two separate *t*-tests, one for each *ilr* log-ratio, were performed to evaluate non-adjusted contributions of each *ilr* to any possible difference between sexes and age groups, using *p*-values as metrics for significance. To further support the interpretation of which behavior in a particular *ilr* explains a possible significant group difference, we developed bootstrap percentile confidence intervals for log-ratio differences between sexes and age groups [29]. The method used to obtain and interpret these intervals is described in Appendix 4.

#### Standard approach

Arithmetic means and standard deviations between workers were calculated for each movement behavior separately (sedentary, standing, PA), stratified by sex and age group. The main effect of sex and age on the proportion of time spent in each movement behavior was determined using two separate MANOVAs. Physical activity time was not included as a dependent variable since the three behaviors add up to 100%. Thus, the proportion of time spent in any behavior can be expressed as a linear combination of the proportions of time spent in the remaining behaviors, resulting in a singular covariance matrix. While we decided to remove PA from the multivariate model, removing sedentary or standing instead would not have changed the results. Additionally, three separate *t*-tests were performed to determine whether each individual behavior differed significantly between sexes and age categories.

Model assumptions in MANOVA and *t*-tests were checked using homogeneity of variance tests and standard graphical procedures. Results obtained with standard and CoDA approaches were considered similar; 1) if the ratio between MANOVA-based η^2^-statistics obtained using the two approaches was close to 1; and 2) if both standard and CoDA results were significant at *P* < 0.05; and 3) if the results of *t*-tests with the *standard* approach were significant at *p* < 0.05, and results of *t*-tests in the CoDA approach were also significant at *p* < 0.05, and the corresponding bootstrap percentile confidence intervals did not include 0. All statistical analyses were performed in the Statistical Package for the Social Sciences (SPSS, version 24), and R (version 3.3.2) using the ‘boot’ [31, 32] and ‘compositions’ packages [33].

## Results

In total, the 677 workers included in the analyses were measured for 29,360 h, with, on average 16.4 (SD 1.4) hours per worker. Details on the recruitment process are shown in Appendix 5. On average, workers were measured for 7.6 (SD1.3) hours at work [men 7.7 (SD 1.3), women 7.5 (SD 1.3), younger 7.6 (SD 1.4), older 7.6 (SD 1.2)] and for 8.8 (SD 1.6) hours during leisure [men 8.6 (SD 1.6), women 9.0 (SD 1.6), younger 8.6 (SD 1.6), older 9.0 (SD 1.6)]. Descriptive statistics of the time spent in each movement behavior (sedentary, standing and PA) obtained with the standard and CoDA approaches are shown in Table 1.

**Table 1:** Descriptive Measures of Percent Time Spent Sedentary, Standing and in Physical Activity at Work and in Leisure According to Standard Analysis (Mean (M), Standard Deviation (SD)) and CoDA (Compositional Mean)

Statistics	Work			Leisure		
	Sedentary	Standing	PA	Sedentary	Standing	PA
	Total (*n* = 677)					
Standard analysis [M(SD)]	31.9(20.7)	49.4(18.2)	18.7(7.4)	62.5(11.7)	27.0(9.2)	10.5(4.4)
CoDA (compositional mean)	29.1	51.3	19.5	63.7	26.2	10.0
	Men (*n* = 370)					
Standard analysis [M(SD)]	35.9(20.5)	46.1(18.9)	18.0(6.9)	64.9(11.3)	25.1(8.8)	10.1(4.4)
CoDA (compositional mean)	33.9	47.1	19.1	66.2	24.3	9.5
	Women (*n* = 307)					
Standard analysis [M(SD)]	27.1(19.8)	53.4(16.5)	19.5(7.9)	59.7(11.5)	29.2(9.2)	11.1(4.3)
CoDA (compositional mean)	24.0	56.2	19.8	60.7	28.7	10.7
	≤45 years (*n* = 312)					
Standard analysis [M(SD)]	33.8(19.8)	47.8(17.9)	18.4(7.6)	61.7(11.9)	27.4(9.3)	10.9(4.6)
CoDA (compositional mean)	31.8	49.2	19.0	62.9	26.7	10.4
	>45 years (*n* = 365)					
Standard analysis [M(SD)]	30.2(21.3)	50.9(18.4)	18.9(7.3)	63.2(11.4)	26.6(9.2)	10.2(4.2)
CODA (compositional mean)	27.0	53.1	20.0	64.4	25.9	9.7

*M* arithmetic mean, *SD* standard deviation, *PA* physical activity (walking, running, cycling, stair climbing), *CoDA* compositional data analysis

The variation matrix (Appendix 3) indicated that behaviors in leisure were, in general, more closely correlated than behaviors at work. The largest variability between workers was observed for ratios at work of sedentary time to stand and to PA.

When comparing geometric means for each sex with the mean of the whole group under the CoDA approach (Appendix 2), differences were observed for all movement behaviors at work and in leisure, i.e., women were less sedentary and more active during both domains than men. When comparing age groups, differences were observed only for sedentary time at work and for PA at leisure, i.e., younger workers were more sedentary at work but more active during leisure than older workers.

Results of Box-M tests showed that the assumption of homogeneity of variances and covariances was met for variables during leisure, but not during work, for both standard and CoDA variables. However, because our sample size was sufficiently large, and the proportion of workers in each strata was almost equal (men 55%, younger 46%), the impact of violating the assumption of equal covariances was considered minimal [34]. Visual inspection of Q-Q plots and histograms of standardized residuals indicated that variables were, in general, normally distributed. Two outliers were identified in boxplots. However, eliminating these outliers from the dataset did not change the results reported below.

When sexes were compared with respect to time spent in different movement behaviors (MANOVA), the η^2^-statistics obtained using the standard approach was 15% smaller than that obtained using CoDA. Both η^2^-statistics were, however, statistically significant at the *p* < 0.05 level. When comparing age groups, η^2^-statistics based on the standard approach was 60% smaller, and not statistically significant, than that obtained with CoDA, which was significant (Table 2).

**Table 2:** Results of Multivariate Analysis of Variance (MANOVA) of Differences in Time Spent Sedentary, Standing and in Physical Activity Between Sexes and Age Groups During Work and Leisure, Analyzed Using Standard and CoDA Approaches

Variable	Work						Leisure					
	Sex			Age			Sex			Age		
	F	η^2^	*p*	F	η^2^	*p*	F	η^2^	*p*	F	η^2^	*p*
CoDA												
*Ilr* coordinates	18.45	0.052	< 0.001	4.21	0.012	0.02	18.19	0.051	< 0.001	2.37	0.007	0.09
Standard analysis												
Sedentary and stand	15.89	0.045	< 0.001	2.62	0.008	0.07	18.36	0.052	< 0.001	2.44	0.007	0.09
Ration of η^2^		1.15			1.60			0.99			0.97	

*CoDA* compositional data analysis, *ilr* isometric log-ratio, *F* test statistic, *p* significance level

During leisure, η^2^-statistics were similar with both approaches, indicating a significant difference between sexes, but a non-significant difference between age groups for time spent in all movement behaviors.

**Fig. 1:**
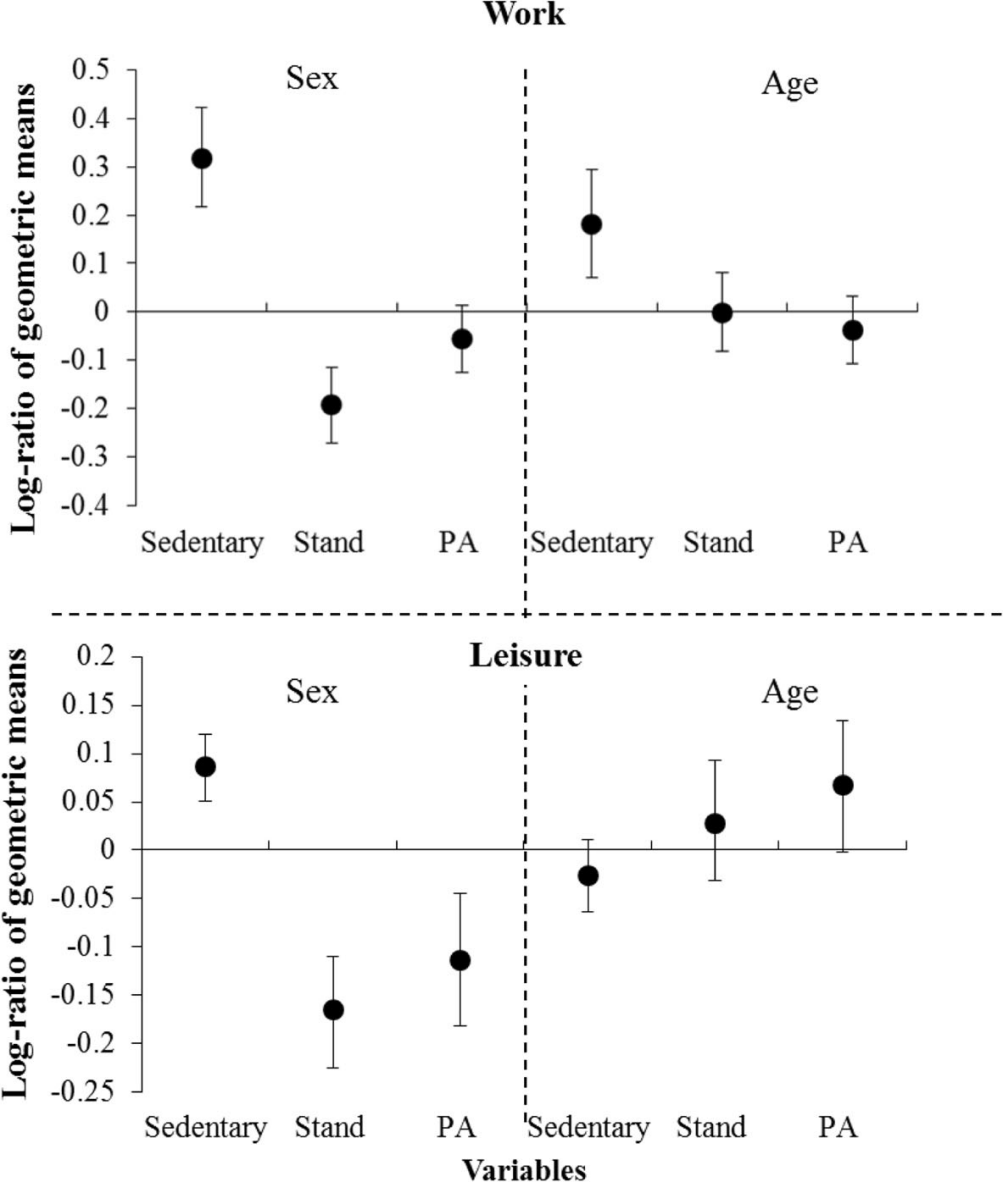
Differences with bootstrap 95% percentile confidence intervals between sexes (left) and age groups (right) in the log-ratio of geometric mean values for sedentary behavior, standing and physical activity. Men/younger was used as the numerator and women/older as the denominator when calculating the log-ratios. Thus, a positive value of the log-ratio indicates that men/younger spent more time in that behavior than women/older workers. A particular behavior is considered significantly different between groups if its confidence interval does not include zero. *PA* physical activity.

The *t*-tests showed that sexes differed significantly during work in both *ilr* coordinates (*ilr*_*1*_: sedentary vs. standing and PA together, *ilr*_*2*_: standing vs. PA). Figure 1 illustrates that men spent more time sedentary and less time standing at work compared to women, while the difference in PA was not significant. Using the standard approach, all three movement behaviors at work, even PA, differed significantly between sexes (Table 3).

**Table 3:** Results of Independent *t*-Tests of Univariate Differences in Time Spent Sedentary, Standing and in Physical Activity Between Sexes and Age Groups During Work and Leisure Analyzed using Standard and CoDA Approaches

Variable	t	MD	CI	*P*	t	MD	CI	*p*
	Sex				Age			
	Work							
ilr_1_	**5.83**	**0.37**	**0.25, 0.50**	**< 0.001**	**2.91**	**0.19**	**0.06, 0.31**	**< 0.001**
ilr_2_	**−3.36**	**−0.10**	**−0.15, −0.04**	**< 0.001**	*−0.52*	*−0.02*	*−0.07, 0.04*	*0.60*
Sedentary	**5.64**	**8.79**	**5.74, 11.84**	**< 0.001**	*2.28*	*3.62*	*0.52, 6.72*	*0.02*
Stand	**−5.38**	**−7.31**	**−9.98, −4.65**	**< 0.001**	*−2.21*	*− 3.08*	*−5.82, − 0.34*	*0.03*
PA	**−2.57**	**−1.48**	**−2.60, − 0.35**	**0.01**	*− 0.95*	*−0.54*	*−1.66, 0.58*	*0.34*
	Leisure							
ilr_1_	**5.67**	**0.18**	**0.12, 0.25**	**< 0.001**	*−1.83*	*−0.06*	*−0.13, 0.00*	*0.07*
ilr_2_	−1.68	−0.04	−0.08, 0.01	0.09	*−1.28*	*−0.03*	*− 0.07, 0.01*	*0.20*
Sedentary	**5.89**	**5.16**	**3.44, 6.88**	**< 0.001**	*−1.72*	*− 1.54*	*−3.30, 0.22*	*0.09*
Stand	**−6.02**	**−4.16**	**−5.53, −2.80**	**< 0.001**	*1.14*	*0.81*	*−0.58, 2.20*	*0.25*
PA	**−2.98**	**−1.00**	**− 1.65, −0.34**	**< 0.001**	*2.19*	*0.73*	*0.07, 1.39*	*0.03*

*CoDA* Compositional data analysis, *ilr*_*1*_ isometric log-ratio coordinate expressing sedentary time vs. standing and PA together, *ilr*_*2*_ isometric log-ratio coordinate expressing standing vs. PA, *PA* physical activity, *t* t-test statistic, *p* significance level, significant results are shown in bold; The faded (italicized) results are for variables which were not significantly different between groups based on MANOVA; *MD* mean difference between groups, *CI* lower and upper limit of a 95% confidence interval on the mean difference

Only *ilr*_*1*_ differed significantly by age (Table 3). According to Fig. 1, younger workers spent significantly more time sedentary at work than older workers. However, the time proportion of standing relative to that of PA (reflected by *ilr*_*2*_) did not differ significantly by age (Fig. 1). In the standard approach, none of the behaviors differed significantly between age groups (Table 3).

During leisure, sexes differed significantly only in the *ilr*_*1*_ coordinate (Table 3), men spending less time standing and in PA, and, thus, more time sedentary than women (Fig. 1). A similar result was obtained using the standard approach. Behaviors during leisure did not differ significantly between age groups, according to neither standard analysis nor CoDA (Table 3).

## Discussion

While the need to use CoDA when analyzing movement behaviors during a day has been highlighted in a number of papers [5, 14–16, 18, 19, 24, 35, 36], CoDA is still rarely used in occupational and public health research. The present paper intends to promote the use of CoDA by explaining the approach in the context of comparisons between groups, and by examining whether time spent sedentary and in physical activity among men and women, and in different age groups stand out differently when using a compositional data analysis (CoDA) compared to using a standard approach. Our study showed that inferential statistics and effect sizes for differences between sexes and age groups do, indeed, depend on the analytical approach. Thus, using CoDA can change the message of a study investigating group differences in time spent on movement behaviors. Our study shows that age and sex are important determinants of how time is used at work and in leisure. The research reported in this paper fits well within the scope of the framework for Viable Integrative Research in Time-Use Epidemiology (VIRTUE) [19]. The VIRTUE framework acknowledges the compositional nature of time-use data and suggests methodological research into addressing differences in effect sizes obtained by standard and CoDA analysis, as well as research examining likely determinants of the composition of physical activity and sedentary behavior.

In the multivariate comparison between age groups, the effect size (partial *eta squared-* η^2^) measuring the overall difference in movement behaviors at work was 60% larger using CoDA than when derived using standard analysis (Table 2). Similarly, when comparing sexes, the multivariate analysis resulted in a 16% larger effect size obtained with CoDA than via the standard appoach. With *p* < 0.05 as the limit for statistical significance, the difference between age groups for time spent in different behaviors was statistically significant according to CoDA, while it was not when using the standard approach. Thus, in a study comparing sexes or age groups with respect to time spent in different movement behaviors, conclusions on statistical significance may differ depending on the analysis approach. Notably, agreement (or not) between the two approaches in regard to whether a group difference shows to be statistically significant may depend on the significance criterion. Had we chosen a significance level of 0.01 or 0.10, the differences between CoDA and standard analyses in terms of statistical significance would have disappeared. In other studies, these specific limits for when results will (dis)agree may obviously be different.

To complete the information offered by the multivariate analysis of differences between groups, we compared groups with respect to each movement behavior using *t*-tests and bootstrap 95% percentile confidence intervals (Fig. 1). The largest disagreement between CoDA and standard analysis appeared when comparing age groups (Fig. 1, Table 2). The univariate analyses confirmed some disagreement between the two approaches in detecting significant differences between age groups in time spent in the three movement behaviors at work.

In leisure, neither standard analysis nor CoDA pointed to any significant differences between age groups or sexes (Table 2). Thus, the two approaches gave similar results in leisure, while at work they did not (Table 2). One reason to this difference between work and leisure could be that the variance in behavior between workers is considerably larger at work that in leisure (Appendix 3). Thus, workers are more likely to show behavior(s) at work occupying close to 0% or 100% of their time than to show equally extreme behaviors in leisure, and this may lead to a more pronounced difference between results under CoDA and standard analyses.

To the best of our knowledge, only one previous study has compared results obtained using CoDA and standard methods [16]. However, in that study, CoDA and standard approaches were used in a regression analysis to investigate the effect of time spent in various behaviors within a day on obesity and cardiorespiratory markers. The study found a difference of about 10 min between arithmetic and compositional group means of moderate-to-vigorous physical activity (MVPA), which is similar to our results of a 12 min difference between CoDA and standard means for PA (Table 1, difference between 18.7 and 19.5% time in PA, corresponding to 12 min). Replacing behaviors associated with a low energy expenditure by just 10 min of MVPA per day can have a significant impact on health outcomes such as obesity [16, 24]. Thus, CoDA and standard analyses may lead to different conclusions, not only from a numeric or statistical viewpoint, but even in terms of the practical applications of study results. In the cited study, the CoDA approach led to attenuated associations, especially for MVPA with cardiorespiratory indicators, compared to the standard approach. In light of these results, and of the findings in the present study, future studies comparing the results of using standard and CoDA approaches in different study designs, and in study populations of various structures appear warranted.

Overall, based on CoDA, we found no difference between age groups in sedentary time and physical activity during leisure, while at work, young workers were more sedentary than older workers. We also found that men were generally less active than women, both during work and leisure, which contradicts previous findings [6, 8, 37, 38]. These differences can be explained by the metric reflecting physical activity used in our study. Previous studies have mainly measured “activity” as the occurrence of vigorous physical activity. In our study, however, “physical activity” included time spent standing, walking, running, stair climbing and cycling. Thus, differences between sexes in physical activity in our study may have occurred in, for instance, walking and not for vigorous physical activities such as running or biking. At work, differences in physical activity between sexes could be due to the job type. In our study population, most cleaners were women while most transport workers were men. Cleaning is associated with extensive standing and moving, while transport workers (preferentially lorry drivers) sit for long periods. Differences between age groups in time spent sedentary compared to other behaviors at work may also relate to job type; the older group comprising more cleaners than the younger group.

In standard analyses of compositional behavior data, any specific behavior is perfectly correlated to the sum of all others. Thus, one variable was removed from the MANOVA model. Most studies dealing with time spent in sedentary behavior and physical activity have not mentioned, let alone addressed, this collinearity issue. The reason may be that behaviors have been expressed in terms of hours/day, not percentages [24, 39], which will not, at a first glance, appear to lead to redundancy issues. A similar issue of the compositional nature of data not being clearly visible appears if single behaviors within a day are analyzed independently in separate univariate analyses. In such scenarios, high correlations between variables may still be present, but the model can be fitted because the compositional nature of data is, to some extent, concealed [40, 41]. Thus, while, standard analyses of compositional data may appear to deliver useful results, they are still basically misleading, since they do not account for the constrained structure of data.

An inherent drawback of the CoDA approach is that essential zeros, such as never spending time on PA in a day, are difficult to handle, since the log-transforms performed as part of the CoDA do not allow zeroes. In the present paper, we avoided essential zeros by merging short durations (which could, in some cases, be zero) of running, cycling, stair climbing and walking into a ‘physical activity’ category. Other ways of dealing with essential zeroes have been suggested, but fall beyond the scope of the present paper [42].

For more than three decades, CoDA has been proposed as the correct approach for analyzing data expressing parts of a whole [10] and CoDA has been implemented to a considerable extent in a number of research areas [43–47]. However, CoDA is still rarely practiced in research devoted to physical activity and sedentary behavior [16–18], or to other biomechanical exposures often expressed as compositions, such as working postures [35]. Emphasizing that results will, in this case, be mathematically correct and correctly interpretable only if analyzed using CoDA, we encourage researchers in occupational and public health to adopt methods and experiences from other disciplines, and apply CoDA in future studies on sedentary behavior and physical activity. This includes studies in populations others than the selection of blue-collar occupations addressed in the present study, and studies devoted to understanding effects of other likely determinants of behavior than sex and age, for instance BMI and musculoskeletal disorders.

### Strengths, limitations and methodological considerations

The major strength of the present study is the access to device-based measures of sedentary behavior and physical activity for more than 29,000 h of work and leisure. Also, data were processed using a validated software, Acti4, which can identify different types of physical activity and body postures with excellent sensitivity and specificity [22].

The main limitation of the study is the inclusion of only blue-collar workers. Since the extent to which results differ between CoDA and standard approaches likely depends on the distribution of data in the investigated population, we emphasize that similar studies should be conducted among other populations, for instance white-collar workers, to validate our findings. Additionally, statistical simulation studies with known differences between groups may also provide valuable insights into a possible bias in effect sizes when using a standard approach compared to CoDA. We also recommend studies to include sleep/bedtime data, since this may influence eventual findings regarding, for instance, differences between sexes and age groups.

## Conclusion

Our results showed that comparisons of sedentary behavior and physical activity between sexes and age groups may lead to different results and, thus, different interpretations depending on whether they are obtained using CoDA or a standard analysis approach, i.e. depending on whether the compositional nature of data is acknowledged or not. We encourage researchers to use CoDA rather than standard analysis when handling compositional data on sedentary behavior and physical activity in occupational and public health studies.

## Figures and Tables

**Fig. 2:**
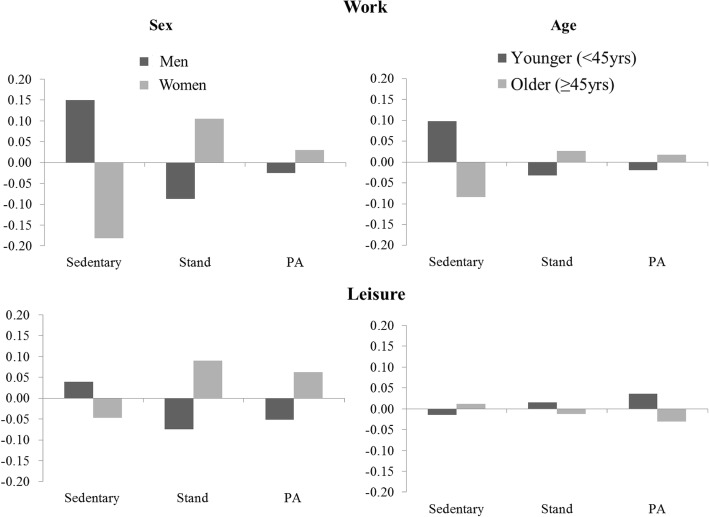
Geometric mean barplot indicating the time spent sedentary, standing and in physical activity (PA) at work and leisure stratified by sex and age, in terms of differences from the geometric mean value of the entire population. Each bar represents the geometric mean of the specific group (for example men), expressed in terms of a ratio measured on a logarithmic scale (as expressed on the y axis) to the geometric mean of the entire population for each behavior (sedentary, standing and PA). A ratio of 0 reflects that the geometric means of the specific group and the entire population are equal. Positive and negative values show that the group geometric mean is larger and smaller, respectively, than the entire population. On the basis of the log ratios displayed in the figures, the actual ratio of the geometric group mean to the whole group geometric mean can be calculated. For example, the bar corresponding to the woman group is negative (−0.18) for sedentary time at work. This means that, on average, women spend 16% (100-exp of −0.18) less time at work in sedentary behavior than the whole group

**Fig. 3:**
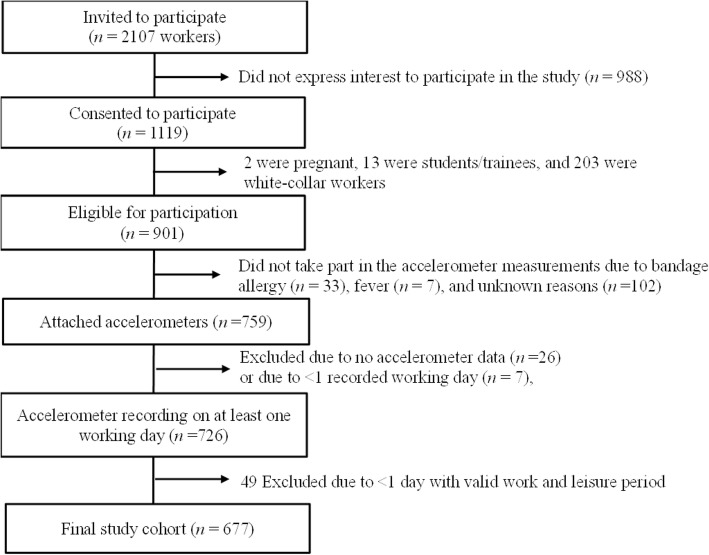
Recruitment process of the participants

## Appendix 1

### Procedure for creating a geometric mean barplot and interpreting it

We assume a compositional data containing three parts *b*_*1*_, *b*_*2*_*,* and *b*_*3*_ (sedentary, standing and PA in our context). The following steps are used to draw a geometric mean barplot:

1. Calculate the geometric mean for each group, *k*, (for example men and women) *g*_*k*_ separately for each part, *b*_*1*_, *b*_*2*_*,* and *b*_*3*_,
2. calculate the overall geometric mean for each part *g* combining all individuals,
3. compute the log-ratio log(*g*_*k*_/*g*), resulting in three log-ratios per group in our case,
4. represent all log-ratios per group as bars in a plot.

If *g*_*k*_ of a part is equal to *g*, then the ratio is 1, the corresponding logarithm is 0 and we do not observe any bar in Appendix 2. If *g*_*k*_ of a part is greater than the *g*, then the logarithm of the ratio is positive and we observe a bar on the positive side. On the contrary, if *g*_*k*_ of a part is lower than the *g*, then the log-ratio is negative and we observe a bar on the negative side. In the barplot, we represent the bars for each group for comparisons.

## Appendix 3

**Table 4:** Variation matrix indicating the dispersion of each movement behavior relative to other movement behaviors within the work and leisure domains

Movement behavior	Sedentary	Stand	PA
	Work		
Sedentary	0	1.32	0.98
Stand	1.32	0	0.30
PA	0.98	0.30	0
	Total variance = 0.86		
	Leisure		
Sedentary	0	0.31	0.33
Stand	0.31	0	0.16
PA	0.33	0.16	0
	Total variance = 0.27		

*PA* physical activity

Numbers in cells show the variance in the data set of the log-ratio between behaviors stated by the row (numerator) and the column (denominator). Values close to 0 indicate that the two behaviors involved are consistently proportional. For example, during leisure, the variance in ln(PA/stand) is 0.16, suggesting that these two behaviors are highly proportional, or co-varying. “Total variance” indicates the total relative variability of the time-use composition during work and leisure. The formula to calculate total variance is presented in Pawlowsky-Glahn & Buccianti [28]

## Appendix 4

### Procedure for making bootstrap percentile confidence intervals for log-ratio differences between groups and interpreting them

The diagram is made using the following steps [29]:

i. The geometric mean of each behavior (%) in both groups are calculated.
ii. The log-ratio of geometric means of both groups (ie., men/women or younger/older) is computed. For sex, the numerator in the log-ratio is men and denominator is women, while for age groups the numerator is younger and the denominator is older workers.
iii. First, 1000 virtual data sets are drawn with replacement from the source population and of the same size. For each resample, the log-ratio of the geometric mean explained in *step ii* is calculated. The resulting distribution of 1000 log-ratios are averaged to calculate bootstrapped mean, and the 2.5th and 97.5th percentiles were selected as upper and lower limits of 95%confidence intervals of the bootstrapped mean.
iv. The resulting bootstrapped mean and their confidence intervals are plotted (see Fig. 1) to determine which behavior of a particular *ilr* contributes to the group differences. If the confidence interval contains the value ‘0’, no difference between the two groups for this particular behavior is identified. Only behaviors for which the intervals are outside 0 are considered responsible for the group differences. Because we use men/young as the numerator and woman/older as the denominator of the log-ratios calculated in step *ii*, a positive value of the log-ratio means that men/young spent more time in that behavior than women/older workers, and *vice versa* if it is a negative value.

## Abbreviations

CoDA: Compositional data analysis
MANOVA: Multivariate analysis of variance
MVPA: Moderate-to-vigorous physical activity
PA: Physical activity
